# Detection of Temporal Clusters of Healthcare-Associated Infections or Colonizations with *Pseudomonas aeruginosa* in Two Hospitals: Comparison of SaTScan and WHONET Software Packages

**DOI:** 10.1371/journal.pone.0139920

**Published:** 2015-10-08

**Authors:** Annick Lefebvre, Xavier Bertrand, Philippe Vanhems, Jean-Christophe Lucet, Pascal Chavanet, Karine Astruc, Michelle Thouverez, Catherine Quantin, Ludwig Serge Aho-Glélé

**Affiliations:** 1 Hospital Epidemiology and Infection Control Department, Dijon University Hospital, Dijon, France; 2 Laboratory of Environmental Microbiology and Health Risks, University of Burgundy, Dijon, France; 3 Infection Control Department, CHU Besançon, Besançon, France; 4 Chrono-environment Laboratory, UMR CNRS 6249, University of Franche-Comté, Besançon, France; 5 Infection Control, Epidemiology and Prevention Department, Hospital Group Edouard Herriot, Lyon, France; 6 Epidemiology and Public Health Team, Claude Bernard University, Lyon, France; 7 UHLIN, Hospital Group Bichat—Claude Bernard, HUPNVS, AP-HP, Paris, France; 8 Paris Diderot University, Paris 7, Paris, France; 9 Infectious Diseases Department, Dijon University Hospital, Dijon, France; 10 Biostatistics and Medical Information Department, Dijon University Hospital, Dijon, France; 11 Epidemiology Department—EA 4184, University of Burgundy, Dijon, France; National Institutes of Health, UNITED STATES

## Abstract

The identification of temporal clusters of healthcare-associated colonizations or infections is a challenge in infection control. WHONET software is available to achieve these objectives using laboratory databases of hospitals but it has never been compared with SaTScan regarding its detection performance. This study provided the opportunity to evaluate the performance of WHONET software in comparison with SaTScan software as a reference to detect clusters of *Pseudomonas aeruginosa*. A retrospective study was conducted in two French university hospitals. Cases of *P*. *aeruginosa* colonizations or infections occurring between 1^st^ January 2005 and 30^th^ April 2014 in the first hospital were analyzed overall and by medical ward/care unit. Poisson temporal and space-time permutation models were used. Analyses were repeated for the second hospital on data from 1^st^ July 2007 to 31^st^ December 2013 to validate WHONET software (in comparison with SaTScan) in another setting. During the study period, 3,946 isolates of *P*. *aeruginosa* were recovered from 2,996 patients in the first hospital. The incidence rate was 89.8 per 100,000 patient-days (95% CI [87.0; 92.6]). Several clusters were observed overall and at the unit level and some of these were detected whatever the method used. WHONET results were consistent with the analyses that took patient-days and temporal trends into account in both hospitals. Because it is more flexible and easier to use than SaTScan, WHONET software seems to be a useful tool for the prospective surveillance of hospital data although it does not take populations at risk into account.

## Introduction

The identification of temporal clusters of healthcare-associated colonizations or infections is a challenge in infection control. Several measures have to be taken to investigate and manage outbreaks of healthcare-associated infections (HAI). The accuracy and rapidity of the detection system is essential to set up appropriate measures. Multidrug resistant (MDR) micro-organisms are usually monitored in healthcare establishments. However, although a daily review of new MDR micro-organisms can be done by simple observation, this is subject to error. Moreover, micro-organisms other than MDR can be sources of outbreaks. SaTScan [1] is a software used to detect clusters of cases. A time trend and the population at risk can be taken into account. The WHO Collaborating Centre for Surveillance of Antimicrobial Resistance developed a software (WHONET/Backlink) for the management of microbiology data, and cluster detection capabilities were developed as an additional analytical feature [2]. WHONET allows SaTScan to run through another interface. Because WHONET is easier to use than SaTScan in daily monitoring of HAI, it could be useful to automatically detect clusters that are not detected with routine surveillance. However, the population at risk cannot be taken into account and it needs to be evaluated in various settings before validation [3]. As part of a study about the role of hospital water system contamination on the incidence of HAI with *P*. *aeruginosa*, we searched for temporal clusters of healthcare-associated colonizations or infections with *P*. *aeruginosa*, overall and by care unit. This retrospective long-term study was conducted in two French university hospitals. This study provided the opportunity to evaluate the performance of SaTScan algorithms implemented in WHONET software in comparison with other algorithms implemented in SaTScan software for the detection of clusters of *P*. *aeruginosa*, in two settings.

## Materials and Methods

The University Hospitals of Dijon and Besançon are located in Burgundy, France and Franche-Comté, France, respectively. They have 1,800 and 1,200 beds, respectively, with medical, surgical and intensive care units.

### Spatial and temporal units

The spatial unit was the care unit. For Dijon Hospital, units that had been joined together or separated during the study period were combined, except for weekly hospitalization units, which were created in 2014 and bring together patients with different risks (cardiology and pneumology, dermatology and rheumatology) and medico-surgical units. The temporal unit was the day.

### Patients

All *P*. *aeruginosa* positive samples for the period 1^st^ January 2005 to 30^th^ April 2014 in Dijon Hospital and for the period 1^st^ July 2007 to 31^st^ December 2013 in Besançon Hospital were extracted from bacteriology laboratory databases using VIGIguard (bioMérieux) software. Duplicates were defined on the basis of the antibiotype and a 6-month period and were excluded using Microsoft Access software (2010). Two isolates were considered different if they were isolated at more than six months apart or if a major difference of antibiotic resistance (one susceptible isolate, one resistant isolate) was observed for one of the following antibiotics: ticarcillin, piperacillin, ceftazidime, imipenem, meropenem, aztreonam, gentamicin, tobramycin, amikacin, ciprofloxacin, and colistin, according to Antimicrobial Committee of the French Society for Microbiology 2013 [4]. Piperacillin, aztreonam, colistin and meropenem were not systematically tested. Patients were also excluded if they had been hospitalized for less than 48 hours at the time the sample was taken, and they were considered localized in the unit where the procedure leading to the first positive sample was prescribed. Microsoft Excel (2010) was used to obtain data in the appropriate format for SaTScan and BacLink was used to format data to be used in WHONET. No consent was required as only anonymous retrospective data from the bacteriology laboratory were used in the study. This study has been registered by the French National Commission for Data Protection and Liberties (CNIL).

### Population at risk

The number of person-days per month was obtained from administrative databases, which record all hospitalizations in both hospitals, and for each care unit. The population at risk was calculated for each day by dividing the number of person-days in the month by the number of days in the month.

### Cluster detection

The Kulldorff scan statistic with a Poisson model was used to detect temporal clusters and the Kulldorff space-time permutation scan statistic was used to detect clusters in space and time [5,6]. Two computer programs: SaTScan [1] and WHONET [2] were used for the retrospective analyses, and simulated prospective analyses were done using WHONET. The analyses were mainly done on Dijon University Hospital data in the context of a study evaluating the role of the hospital water system in the occurrence of clusters of *P*. *aeruginosa*. Besançon University Hospital data were used to evaluate WHONET software compared with SaTScan in another setting. The analyses were first conducted at the whole hospital level for Dijon Hospital and then at the unit level. Analyses were conducted at the unit level only for Besançon hospital.

SaTScan software made it possible to adjust for secular log linear trends, day of the week, and population at risk (number of patient-days) through a retrospective temporal scan, assuming a Poisson distribution while WHONET software did not. Indeed, SaTScan was considered a reference to evaluate the performance of WHONET software. *P values* were obtained with retrospective analyses whereas recurrence intervals were obtained with prospective analyses. The recurrence interval is the inverse of the *P value*. If a recurrence interval is 365 days, then under the null hypothesis of no cluster, such a cluster could have been observed by a random chance every 365 days. A *P value* of 0.05 and a recurrence interval of 365 days were used as the threshold to identify a statistical cluster. Analyses were repeated by adjusting for the clusters detected [7] with a maximum cluster duration of 1,000 days, with 999 iterations for Monte Carlo simulations. Sensitivity analyses were done by setting the maximal duration of related clustered cases at 120 days. To avoid repeating unit-by-unit analyses, temporo-spatial scans were done by limiting the size of the cluster in terms of maximal distances between units, after entering fictitious coordinates for each unit. This avoided including more than one unit in each cluster. Analyses were adjusted for relative risk in each unit.

Monte Carlo simulations were performed using a Poisson distribution for the global detection of temporal clusters in WHONET. Space-time permutation models were used for analyses by unit. Sensitivity analyses were conducted for simulated prospective analyses with reference periods of 1, 2 or 4 years. Analyses with a reference period of 2 and 4 years were performed on data since 2007 and 2009, respectively, for Dijon Hospital and on data since July 2009 and July 2011, respectively, for Besançon Hospital. Retrospective analyses took the whole period into account.

We compared the results obtained with the various methods: (1) retrospective analyses without adjustments (WHONET), (2) prospective analyses without adjustment, with reference periods of one, two and four years (WHONET), (3) retrospective analyses taking the population at risk into account (SaTScan 1), and (4) retrospective analyses taking the population at risk, the time trend and the day of the week into account (SaTScan 2). Data of both University Hospitals were analyzed independently. Analyses by resistance profiles were also done by care unit.

Resistance profiles were investigated when clusters by care unit were detected even though no clusters were detected by resistance profiles in the same unit and the same period. Finally, the probability (low, medium or high) that further investigations would have been conducted if the infection control ward had been aware of the alert was also evaluated for the clusters detected in the analyses by care unit at the Dijon hospital level.

## Results

At Dijon University Hospital, during the study period, 3,946 isolates of *P*. *aeruginosa* were recovered from 2,996 patients, cumulating 4,395,596 patient-days (S1 Dataset). The incidence rate was 89.8 per 100,000 patient-days (95% CI 87.0; 92.6). Analysis of the data for the 2,996 patients showed a mean age of patients of 66.0 years (SE 0.4 years) at the time of the first positive sample. The median age was 70 years (interquartile range [57–81]). Forty-two percent were women. Most patients had one isolate (2,396 patients or 80%), 395 (13%) patients had two isolates, 125 (4%) had 3 isolates and 80 (3%) had at least 4 isolates.

**Fig 1:**
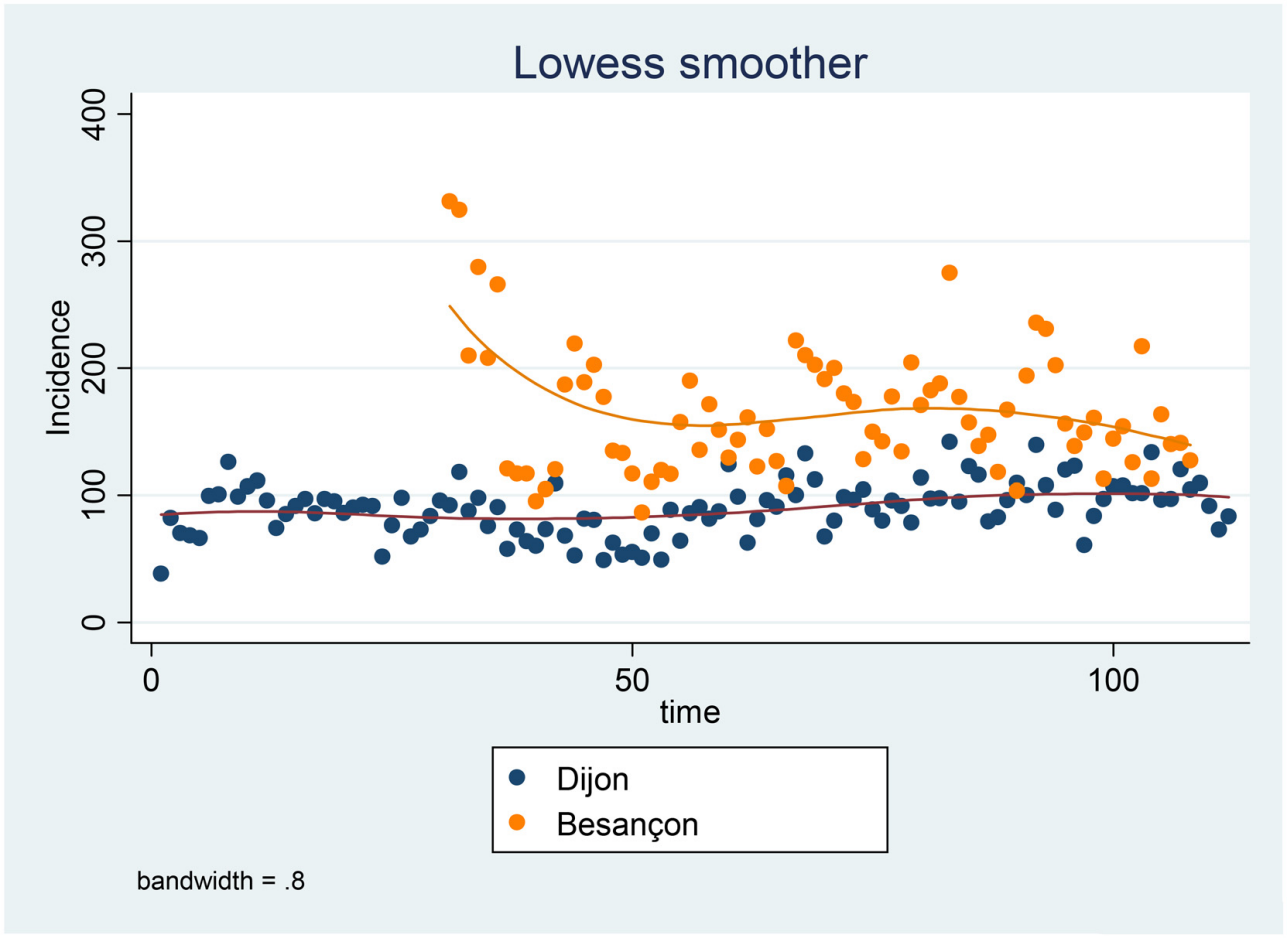
Evolution of incidence (per 100,000 patient-days) of *P*. *aeruginosa* colonizations or infections at Dijon and Besançon Hospitals by month between January 2005 and April 2014.

An overall increase in incidence of 2.63% per year was detected with SaTScan. The incidence by month per 100,000 patient-days is shown in Fig 1. Poisson regression of incidence by month showed that this increase was significant (p<0.001).

**Fig 2:**
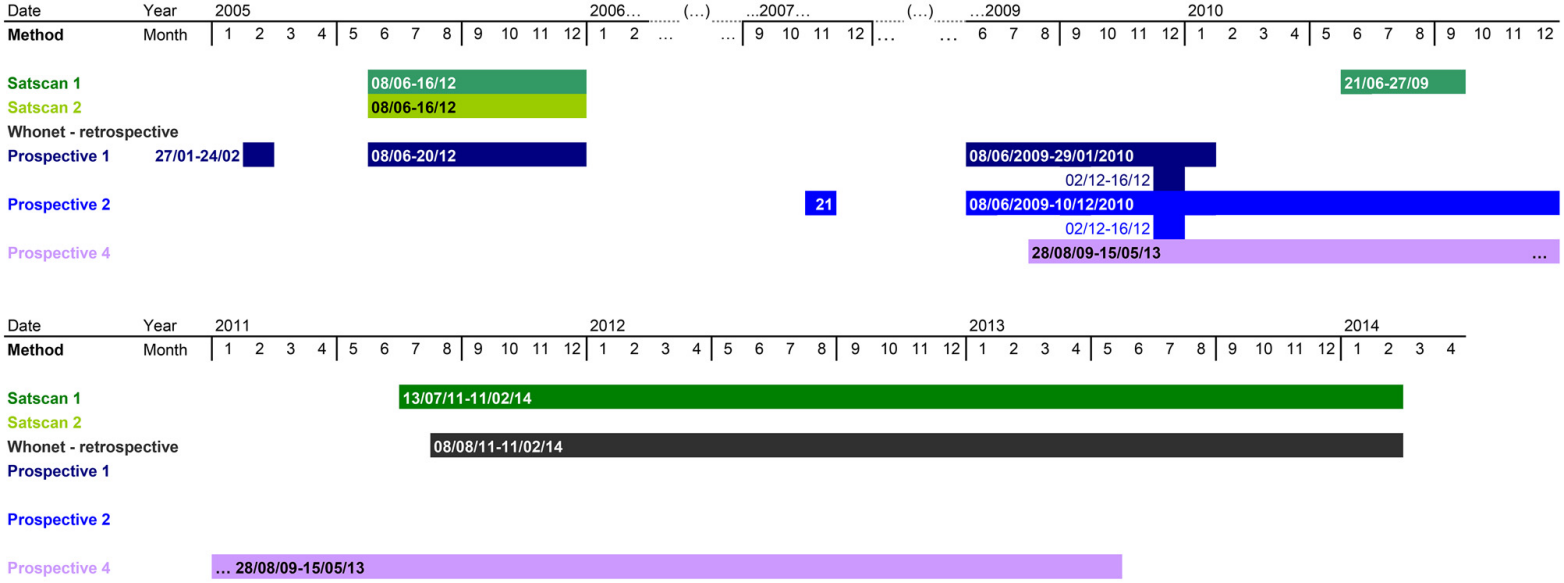
Distribution of statistical clusters according to the method for Dijon University Hospital between January 2005 and April 2014. SaTScan 1: Temporal Poisson, adjustment for population at risk; SaTScan 2: Temporal Poisson, adjustment for population at risk, on linear trend of 2.628% per year and day of the week; WHONET Retrospective Temporal Poisson, no adjustment; Prospective 1, 2 and 4: simulated prospective space-time permutation (WHONET), reference period respectively of 1, 2 and 4 years.

For the hospital overall, several statistical clusters were detected or not depending on the method/software used (tables 1 and 2; Fig 2), and some were detected with several methods. The dates and number of clusters varied depending on the method used.

**Table 1:** Retrospective statistical temporal clusters detected at Dijon University Hospital overall with SaTScan (adjusted for population at risk) and WHONET between January 2005 and April 2014 using the Kulldorff statistic with Poisson Monte Carlo simulation.

Software/ Method	Cluster start	Cluster end	P value	Observed cases	Expected cases	Observed/Expected
**SaTScan (maximal size of 1000 days)**	13/07/2011	11/02/2014	0.001	1322	1129.21	1.17
	21/06/2010	27/09/2010	0.023	150	100.44	1.52
	08/06/2005	16/12/2005	0.005	258	190.32	1.36
**SaTScan (maximal size of 120 days)**	27/05/2005	10/10/2005	0.014	157	105.39	1.49
**SaTScan with temporal linear trend (increase of 2.628% per year) and on day-of-week adjustments (maximal size of 1000 days)**	08/06/2005	16/12/2005	0.005	258	185.67	1.39
**SaTScan with temporal linear trend (increase of 2.628% per year) and on day-of-week adjustments (maximal size of 120 days)**	19/07/2012	11/09/2012	0.012	105	63.13	1.66
**WHONET (maximal size of 1000 days)**	08/08/2011	11/02/2014	0.001	1292	1064.7	1.21
**WHONET (maximal size of 120 days)**	-	-	-	-	-	-

**Table 2:** Statistical temporal clusters detected with WHONET in Dijon University Hospital overall between January 2005 and April 2014, simulated prospective temporal Poisson.

Software/ Method	Cluster start	Cluster end	Recurrence interval	Observed /expected cases*	First detection
**WHONET, reference 1 year**	**27/01/2005**	24/02/2005	1000 days	33/21 from 29/01 to 24/02	01/02/2005
	**08/06/2005**	20/12/2005	1000 days	233/194 from 29/06 to 20/12	23/08/2005
	**08/06/2009**	29/01/2010	1000 days	204/170 from 12/06 to 10/12	18/11/2009
**WHONET, reference 2 years**	**21/11/2007**	21/11/2007	500 days	7/1.1	21/11/2007
	**02/12/2009**	16/12/2009	1000 days	30/14.1	13/12/2009
**WHONET, reference 4 years**	**08/06/2009**	10/12/2010	1000 days	340/288.86 from 16/03 to 10/12	30/12/2009
	**02/12/2009**	16/12/2009	1000 days	30/14.1	13/12/2009
	**28/08/2009**	15/05/2013	100000 days	899/819 from 08/08/2011 to 15/05/2013	30/06/2010

* Beginning of cluster can move with detection date.

Retrospective analysis of Dijon Hospital data using WHONET software highlighted a single overall cluster. The beginning and the end of this cluster were included in the first cluster detected by SaTScan without taking time trends into account (August 8^th^, 2011 to February 11^th^, 2014 and July 13^th^, 2011 to February 11^th^, 2014, respectively). This cluster was not detected when the time trend was taken into account.

Retrospective analyses taking account of both the population at risk and the time trend (with SaTScan) as well as simulated prospective scans with Poisson distribution Monte-Carlo simulation (with WHONET) detected the first cluster during the study period, from June to December 2005. However, retrospective analyses with WHONET did not detect this cluster. A third cluster detected with SaTScan without taking the time trend into account occurred between June 21^st^, 2010 and September 27^th^, 2010. This cluster was not detected using other retrospective analysis methods. The simulated prospective temporal scan allowed the detection of this cluster.

Another cluster, during February 2005 (27/01/2005 to 24 or 25/02/2005 depending on the reference period), was identified with simulated prospective temporal scans. Other clusters were not detected with at least one reference period used in simulated prospective scans. One cluster was identified during 2009–2010. It lasted six months and one and a half years with reference periods of one and two years, respectively. The second contained one cluster identified with SaTScan (June to September 2010).

Analyses by unit allowed us to detect 12 statistical clusters (table 3). Two of these were detected in newly created units. Two others were detected with retrospective analysis using both WHONET and SaTScan, with the same start and end times for one of them (cluster number 10) and a later start date for the other (cluster number 5: 16/03/2010 to 05/07/2010 with SaTScan and 21/02/2010 to 05/07/2010 with WHONET). The first cluster detected using SaTScan with adjustment for the temporal trend and day of the week was not detected with other methods. Simulated prospective scans using WHONET identified several clusters in units other than newly created units. One cluster was detected regardless of the reference period (2 cases in three days, cluster number 2). A second cluster (number 5, with 3 cases) was identified with all three reference periods tested. This cluster was not identified with SaTScan. Other clusters were detected according to only one of the three reference periods tested. One of them had a recurrence interval of 2103 days. The others had a lower recurrence interval. In four cases, a cluster was also detected using resistance profile analyses. The probability that the cluster would have been investigated if the infection control unit had been aware of the cluster outside this context was low in 3 of 9 cases, medium in 5 cases and high in one case that was partly identified by the bacteriology laboratory and was investigated. The alert would have occurred at the time of the occurrence of the 23^rd^ of 54 cases with prospective analysis with a reference period of 4 years, and at the time of the occurrence of the 38^th^ of 44 cases using a reference period of 2 years. In other cases, with a medium probability that the cluster would have been investigated, the investigations would not have been conducted before the last case of the cluster because the first report did not occur until 2 days before the last case. Analyses by resistance profile led to the identification of three other clusters, with a retrospective method (not shown). Several other clusters were detected with prospective analyses by resistance profile, but they involved only two cases (7 clusters with a reference period of 1 year or 2 years and 8 clusters with a reference period of 4 years).

**Table 3:** Statistical temporal clusters by care unit according to the method in simulated prospective analyses for Dijon University Hospital between January 2005 and April 2014.

Cluster number	Software/methods detecting this cluster	Year	Recurrence interval	P value	Duration	Observed/ expected cases	Detection of clusters by resistance profile	Same resistance profile when investigating the cluster	Probability that cluster would have been investigated if aware of the cluster
1	**SaTScan 2**: temporal Poisson adjusted for population at risk, days-of-week and temporal trend	2005	-	0.011	4 months	18/3.55 = 5.07	No	No	Medium
2	**WHONET: Simulated prospective** space-time permutation, reference period: 1 year	2006	1192 days	-	3 days	2/0.014 = 143	Yes	-	Medium
3	**WHONET: Simulated prospective** space-time permutation, reference period:1 year	2007	371 days	-	2 days	5/0.47 = 10.6	Yes but different cluster dates	No (2 patients– 5 different resistance phenotypes	Low
4	**WHONET: Simulated prospective** space-time permutation, reference periods: 1, 2 and 4 years	2009	19219, 9282 and 4714 days, respectively	-	1 day	3/0.038 = 7.9, 3/0.035 = 8.6, 3/0.032 = 9.4	No	No (2 patients—3 different resistance phenotypes)	Low
5	**SaTScan 1**: temporal Poisson adjusted for population at risk	2010	-	0.007	4 months	40/14.42 = 2.8	Yes (10 cases)	-	High—proved cross transmission
	**SaTScan 2**: temporal Poisson adjusted for population at risk, days-of-week and temporal trend		-	0.012	4 months	40/14.58 = 2.7			
	**WHONET: retrospective** space-time permutation		-	0.0023	4 months	43/16.48 = 2.6			
	**WHONET: Simulated prospective** space-time permutation, reference periods: 2 and 4 years		2226 and 182071 days, respectively	-	4 and 8 months	44/22.3 = 2.0, 59/31.37 = 1.9			
6	**WHONET: Simulated prospective** space-time permutation, reference period: 2 and 4 years	2010–2011	610 and 462 days, respectively	-	8 days	4/0.18 = 22.2, 4/0.17 = 23.5	No	No	Medium
7*	**WHONET: retrospective** space-time permutation	2011	-	<0.0001	7 months	44/15.27 = 2.9	No	No	Low
	**WHONET: Simulated prospective** space-time permutation, reference period: 4 years	2011–2013	311787 days	-	9 months	15/2.97 = 5.05			
8*	**WHONET: retrospective** space-time permutation	2011–2012	-	0.004	5,5 months	12/1.51 = 7.9	No	No	Low
	**WHONET: Simulated prospective** space-time permutation, reference period: 4 years		816759 days	-		12/1.70 = 7.1			
9	**WHONET: Simulated prospective** space-time permutation, reference periods: 1 and 4 years	2012	381 and 43988 days, respectively	-	3 weeks	6/0.65 = 9.2, 6/0.57 = 10.5	No	No	Low (one patient had four different strains—five screening digestive samples and one blood culture)
10	**SaTScan 1**: temporal Poisson adjusted for population at risk	2012	-	0.017	3 weeks	9/0.72 = 12.5	Yes (3 cases)	-	Medium
	**SaTScan 2**: temporal Poisson adjusted for population at risk, days-of-week and temporal trend		-	0.045		9/0.78 = 11.5			
	**WHONET: retrospective** space-time permutation		-	0.039		9/0.96 = 9.4			
	**WHONET: Simulated prospective** space-time permutation, reference periods: 2 and 4 years		810 and 1787 days, respectively	-		9/1.38 = 6.5, 9/1.07 = 8.4			
11	**WHONET: Simulated prospective** space-time permutation, reference period: 1 year	2013	2103 days	-	6 days	3/0.057 = 53	No	No– 2 patients– 3 resistance phenotypes	Low
12	**WHONET: Simulated prospective** space-time permutation, reference period: 2 years	2014	412 days	-	7 days	3/0.077 = 39	No	Two of three with same resistance profile	Medium

p value are given for retrospective analyses and maximum recurrence interval are given for prospective analyses.

* unit created in 2011.

At Besançon University Hospital, 3,467 isolates of *P*. *aeruginosa* were recovered for 2,184,332 patient-days (S2 Dataset). The incidence was thus 159 [156–164] per 100,000 patient-days. Twenty temporal statistical clusters were detected. Most of these were detected with various methods (table 4). The probability that the cluster would have been investigated was low except for six of them (medium in five cases: clusters number 9, 11, 13, 16 and 18, and high in one case: cluster number 4). The alert would have occurred before the end of the clusters in three cases. Concerning cluster number 4, which lasted 4 months, the alert would have occurred after ten days. Concerning cluster number 16, the alert would have occurred nearly three days before the end of the cluster, which lasted three weeks. Concerning cluster number 18, the alert would have occurred nearly ten days before the end of the cluster, which lasted three months. Analyses by resistance profile led to the identification of ten other statistical clusters (one cluster with 5 cases, three clusters with 3 cases, and six with two cases).

**Table 4:** Temporal clusters by unit according to the method for Besançon University Hospital between July 2007 and December 2013.

Cluster number	Software/methods detecting this cluster	Year	Duration	Recurrence interval (days)	P value	Observed/ expected cases
1	**WHONET: retrospective** space-time permutation	2007	1.5 months		0.00809	73/37.14 = 2.0
	**SaTScan 1**: temporal Poisson adjusted for population at risk		1 month		<0.001	51/15.08 = 3.4
	**SaTScan 2**: temporal Poisson adjusted for population at risk, days-of-week and temporal trend		1 month		<0.002	52/16.18 = 3.2
2	**SaTScan 1**: temporal Poisson adjusted for population at risk	2007	3 months		<0.001	63/22.33 = 2.8
	**SaTScan 2**: temporal Poisson adjusted for population at risk, days-of-week and temporal trend				<0.002	64/24.08 = 2.7
3	**SaTScan 1**: temporal Poisson adjusted for population at risk	2007	5.5 months		<0.001	65/23.92 = 2.7
	**SaTScan 2**: temporal Poisson adjusted for population at risk, days-of-week and temporal trend				<0.001	65/25.92 = 2.5
4	**WHONET: Simulated prospective** space-time permutation, reference period: 1 year	2008	4 months	5079		16/0.17 = 94.1
5	**WHONET: Simulated prospective** space-time permutation, reference period: 1 year	2008	11 days	855		3/0.08 = 37.5
6	**WHONET: Simulated prospective** space-time permutation, reference period: 1 year	2009	9 days	377		3/0.079 = 38.0
7	**SaTScan 1**: temporal Poisson adjusted for population at risk	2009	11 days		0.022	5/0.11 = 45.5
	**SaTScan 2**: temporal Poisson adjusted for population at risk, days-of-week and temporal trend				0.034	5/0.12 = 41.7
	**WHONET: retrospective** space-time permutation				0.042	5/0.17 = 29.4
	**WHONET: Simulated prospective** space-time permutation, reference period: 2 years			20197		5/0.18 = 27.8
	**WHONET: Simulated prospective** space-time permutation, reference period: 1 year			14302		5/0.24 = 20.8
8	**WHONET: Simulated prospective** space-time permutation, reference period: 2 years	2010	6 days	446		13/3.36 = 3.9
	**WHONET: Simulated prospective** space-time permutation, reference period: 1 year			720		13/2.23 = 5.8
9	**WHONET: Simulated prospective** space-time permutation, reference period: 2 years	2010	8 days	1885		4/0.059 = 67.8
10	**WHONET: Simulated prospective** space-time permutation, reference period: 2 years	2010	3 days	396		4/0.2 = 20.0
	**WHONET: Simulated prospective** space-time permutation, reference period: 1 year			700		4/0.21 = 19.0
11	**WHONET: Simulated prospective** space-time permutation, reference period: 2 years	2011	3 days	402		3/0.067 = 44.8
12	**WHONET: Simulated prospective** space-time permutation, reference period: 2 years	2011	3 days	1527		3/0.046 = 65.2
	**WHONET: Simulated prospective** space-time permutation, reference period: 1 year			1129		3/0.014 = 214.3
	**WHONET: Simulated prospective** space-time permutation, reference periods: 4 years			443		3/0.057 = 52.6
13	**WHONET: Simulated prospective** space-time permutation, reference period: 2 years	2012	7 days	1089		7/0.77 = 9.1
	**WHONET: Simulated prospective** space-time permutation, reference period: 1 year			4640		7/0.7 = 10.0
14	**WHONET: Simulated prospective** space-time permutation, reference period: 1 year	2012	3 days	709		2/0.01 = 200.0
15	**SaTScan 1**: temporal Poisson adjusted for population at risk	2012	2 days		0.022	4/0.043 = 93.0
	**SaTScan 2**: temporal Poisson adjusted for population at risk, days-of-week and temporal trend				0.0053	4/0.031 = 129.0
	**WHONET: Simulated prospective** space-time permutation, reference period: 2 years			5913		4/0.1 = 40.0
	**WHONET: Simulated prospective** space-time permutation, reference period: 1 year			14598		4/0.099 = 40.4
	**WHONET: Simulated prospective** space-time permutation, reference periods: 4 years			3690		4/0.1 = 40.0
16	**WHONET: Simulated prospective** space-time permutation, reference period: 2 years	2012	3 weeks	712		11/1.82 = 6.0
	**WHONET: Simulated prospective** space-time permutation, reference periods: 4 years			1048		11/1.67 = 6.6
	**SaTScan 2**: temporal Poisson adjusted for population at risk, days-of-week and temporal trend	2012	6 months		0.036	38/14.27 = 2.7
17*	**WHONET: retrospective** space-time permutation	2012	14 days		0.044	3/0.02 = 150.0
	**WHONET: Simulated prospective** space-time permutation, reference period: 2 years			410		3/0.06 = 50.0
	**WHONET: Simulated prospective** space-time permutation, reference periods: 4 years			2386		3/0.032 = 93.8
18	**WHONET: Simulated prospective** space-time permutation, reference periods: 4 years	2013	3 months	561		45/20.26 = 2.2
19	**WHONET: Simulated prospective** space-time permutation, reference period: 1 year	2013	11 days	570		4/0.23 = 17.4
20	**WHONET: Simulated prospective** space-time permutation, reference period: 1 year	2013	1 day	481		2/0.016 = 125.0

* unit created in 2012

## Discussion

### Interpretations

This long-term study involved all cases of HAI or colonizations with *P*. *aeruginosa* occurring in two hospitals. Even though different clusters were detected during the study period, with slightly different results depending on the method used, the results obtained with WHONET and SatTScan were consistent as most of the clusters were detected by both programs.

#### Performances

SaTScan software was used to search for clusters either directly or through WHONET software. SaTScan software had the advantage of taking the population at risk, a possible time trend, and the day of the week into account. Retrospective analysis of Dijon Hospital data using WHONET software highlighted a single overall cluster included in the first cluster detected by SaTScan without taking time trends into account and overlapped the cluster detected with simulated prospective analysis with a reference period of four years. This cluster was not detected when the time trend was taken into account or with simulated prospective analysis with periods of one or two years. This cluster was wide and probably reflected the overall trend, which was not taken into account with the Poisson distribution Monte-Carlo simulation in WHONET and had a lesser impact in analyses with short reference periods. This temporal trend could be due to increased comorbidity in patients, for example, or to increased exposure to *P*. *aeruginosa* in the water system. In the latter case, we would expect a simultaneous increase in other waterborne micro-organisms. However, we did not analyze data about other micro-organisms. It can also be a spurious signal related to the large volume of data. Indeed, further evaluation and further analyses of the resistance profile showed no particular resistance phenotype, but an increase in sensitive strains only during this period. The space-time permutation model takes purely temporal variations into account (as purely spatial variations) [6]. However, this method could not be used for temporal analyses at the whole hospital level as there was only one spatial unit.

The cluster observed in February 2005 with simulated prospective temporal scan, with a sudden increase in incidence, was probably due to sampling fluctuations. It was observed following a particularly low incidence in January 2005, the beginning of the study period. More importantly, a very short reference period (27 days) was taken into account and did not allow the use of a one-year reference period. This cluster was not detected with retrospective methods, except with SaTScan adjusted for time trends when the maximal duration of the cluster was set at 120 days.

Searching for clusters per care unit using WHONET software led to the identification of two types of clusters: those due to the creation of care units without taking the population at risk into account, and those detected outside this context. The probability that a cluster would have been investigated if the infection control unit had been aware of the cluster outside this context was low in only 3 of 9 cases. In one case, the cluster has been partly identified by the bacteriology laboratory. Indeed, an alert was issued for clusters of *P*. *aeruginosa* in respiratory samples from the medical ICU. This outbreak was connected to the use of the same contaminated endoscope in these patients. The strain in the endoscope and in four patients was the same as that identified by genotyping. The infection control unit did not investigate the other cases (40 or 54 cases in total depending on the method) included in the clusters although 10 of them had the same resistance profile because the outbreak had not been detected. All of the algorithms except the simulated prospective analysis using one year as a reference period detected this outbreak. Analyses were also done at the unit level on data from Besançon University Hospital to validate the method in another setting, with a higher incidence of *P*. *aeruginosa* cases. The results for retrospective analyses, whether the time trend was taken into account or not, and for simulated prospective analyses were consistent in both hospitals. Clusters detected with SaTScan at the beginning of the period could not be detected with prospective simulation due to the lack of data before the cluster. Other clusters were detected with most of the prospective simulations. Additional clusters were detected in prospective simulations with only one algorithm. In these cases, the maximum recurrence intervals were generally lower (under 1000 days).

When the time trend was taken into account with SaTScan, the clusters detected in Dijon Hospital at the hospital level were different for the two programs. However, when it was not taken into account with SaTScan, the results were consistent with the retrospective analysis using WHONET, which cannot take account of either the population at risk or linear time trends in temporal Poisson regressions. The simulated prospective search with the previous year or two years serving as a reference reduced the influence of time trend. The population at risk seemed to have a lesser influence on the results than did the temporal trend. Analyses per unit for clusters in units other than newly created units were consistent. The space-time permutation method in WHONET makes it possible to take account of seasonal or day-of-the-week variations occurring simultaneously in all of the “spatial units” analyzed. Spatial units can be care units or resistance profiles, for example. Indeed, the software also allows the detection of clusters of cases by resistance profiles.In Besançon Hospital, the results were globally consistent, especially when the recurrence interval was high.

#### Usability of the softwares

SaTScan software allows spatiotemporal analyses that consider connections between spatial units (first nearest neighbor, second nearest neighbor…). This analysis cannot be run with WHONET. However, clusters concerning only a group of care unit can be searched for by using the ward grouping option to consider care units of the same floor, or the same ward, or the same type together, for example. The latitude and longitude of spatial units can also be considered to search for spatio-temporal clusters, but this is not relevant for hospital-based detection. Data management is relatively easy with WHONET software, which includes a subset of SaTScan’s capabilities that were optimized and considered most relevant for microbiology laboratories. WHONET is easier for routine clinical laboratory staff to use as it does not require an advanced background in statistics and database programming. Furthermore, the results can be presented easily in standard WHONET output tables and charts.

Both softwares allow retrospective analyses that address the question of the occurrence of clusters during a study period (“did we have an outbreak last year?”). Prospective analyses, searching for current clusters for each day of the period, are also possible with both softwares, thus allowing the detection of outbreaks in real-time. Even though the alert occurred at the end of the statistical cluster in several cases, it occurred before the end of the cluster in several other cases. In the latter situation, if the infection control department had been aware of the alert, measures could have been taken to reduce the duration of the outbreak. WHONET software also allows simulated prospective analyses, which address the question of the clusters that we would have detected if we had run daily prospective analyses during the study period. These analyses are not possible with SaTScan unless data management and analyses are automated.

### Strengths and limitations

To our knowledge, few large-scale, long-term studies of clusters of HAI or colonizations have been published. Moreover, our study was conducted in two university hospitals, with different settings and different incidences, in which we included all cases in both hospitals over a long period.

The clusters were investigated with several methods based on the Kulldorff space-time permutation model and on the Poisson temporal scan. SaTScan was used directly or through WHONET software, and we conducted sensitivity analyses. The clusters were investigated over a long period but relatively few clusters were detected. This can be explained by the use of the Kulldorff statistic, which takes into account multiple testing, thus avoiding the over-detection of clusters [5]. The Kulldorff statistic also allows covariables to be taken into account.

One of the strengths of our study was to be conducted in two university hospitals, in the long term, with different settings and different incidences, in which we included all cases in both hospitals over a long period. The aim was not to compare the two hospitals but to validate the method on data from a second hospital.

Simulated prospective analyses using WHONET made it possible to obtain data about the clusters that we would have observed in real-time if we had used WHONET in the daily monitoring of laboratory databases. These automatic methods allowed the detection of clusters not detected before the implementation of these methods. Thus, the infection control department was aware of only one cluster of colonization or infection with *P*. *aeruginosa* detected during the period by the microbiology laboratory.

The methods used here are applicable to the prospective detection of clusters of healthcare-associated infections in other settings. Given its ease of use for repeated tests over time, WHONET software can be a useful tool to perform these analyses, provided that the population in each care unit does not disproportionately increase or decrease compared with others.

This study had several limitations. Firstly, the detection of clusters itself can be controversial. Indeed, cases are not systematically connected. Clusters occurring at the hospital level in Dijon University Hospital are difficult to interpret because this Hospital is spread over several sites. The role of cross-contamination or contamination of the water system in the simultaneous occurrence of clusters in different sites seems unlikely. Only temperature conditions, for example, or low water flow from outlets in several units at the same period (periods of bed closures), could favor the development of a biofilm in water systems of all buildings in the same period. Secondly, only two software packages, both using a temporal scan statistic with different adjustments and simulation methods (Poisson Monte Carlo replications with SaTScan and analyses at the hospital level with WHONET and space-time permutations in other analyses using WHONET) were tested. WHONET software is easy to use in the search for clusters in the daily monitoring of HAI, but it does not take the population and the time-trend into account. This is why we chose to study the performance of WHONET in comparison with a software (SaTScan) that uses the same methods but allows these adjustments. Finally, the detection of clusters focused on *P*. *aeruginosa* only because this study was part of a study about the association between hospital water system contamination and HAI or colonizations. Indeed, this organism is frequently found in water systems and is subject to regulatory research. In addition, it is frequently involved in nosocomial infections.

## Conclusion

Our work is the first to investigate clusters of HAI or colonizations due to *P*. *aeruginosa*, possibly due to water contamination, in two university hospitals, with different settings and different incidences, over a long period.

The results obtained with WHONET and SaTScan were consistent. They allowed the identification of clusters not detected without automatic methods. WHONET seems to be an interesting tool for the surveillance of HAI although it does not take the population at risk into account. A reference period of one or two years can be used.

## Supporting Information

S1 Dataset Dijon minimal dataset. S: susceptible, I: intermediate, R: resistant.
(XLS)

S2 Dataset Besançon minimal dataset. S: susceptible, I: intermediate, R: resistant.
(XLS)
